# Establishing and Comparing the Normal apparent Diffusion Coefficient Values of Fetal Organs and Placenta Using 1.5 Tesla and 3.0 T MRI at Various Gestational Age

**DOI:** 10.4314/ejhs.v33i4.8

**Published:** 2023-07

**Authors:** Priyanka Chandrasekhar, Rajeswaran Rangasami, Chitra Andrew, N. Paarthipan

**Affiliations:** 1 Department of Radiology, Sri Ramachandra Institute of Higher Education and Research, Chennai-600116, India; 2 Department of Fetal Medicine,Sri Ramachandra Institute of Higher Education and Research, Chennai-600116, India; 3 Department of Radiology, Saveetha Medical College and Hospital,Chennai-602105, India

**Keywords:** Diffusion-weighted imaging (DWI), apparent diffusion coefficient (ADC), fetal MRI, field strength

## Abstract

**Background:**

Diffusion-weighted imaging (DWI) is the random Brownian motion of water molecules within a tissue voxel. The apparent diffusion coefficient (ADC) is a quantitative parameter calculated from the DWI that directly reflects the mobility of water molecules in biological tissues. The objective of this study was to establish and compare the normal reference ADC values of fetal organs and the placenta using 1.5 T and 3.0 T MRI at various gestational ages.

**Methods:**

This was a retrospective and prospective observational study. This study included one hundred and three (103) singleton pregnancies for each magnetic field strength. Diffusion-weighted imaging was performed using single-shot spin-echo-planar imaging (EPI) in the axial plane of the fetal head-trunk with a slice thickness of 4mm and diffusion gradient values of b = 0 and b = 700–800 s/mm^2^.

**Results:**

The mean ADC values of cerebral WM areas were significantly higher than the deep grey areas in the brain. The white-matter regions, lung, and placenta showed a positive and significant correlation with increasing gestational age in both field strengths. A statistically weak negative correlation was observed between increasing gestational age and ADC measurements obtained in the thalamus, cerebellum, pons, and kidney.

**Conclusion:**

This study gives the reference values for both 1.5T and 3T MRI of vital organs. The current study shows that diffusion-weighted MRI can offer a promising technique to evaluate the structural development of fetal organs and can potentially act as a biomarker for predicting the functionality of the fetal organs in abnormalities.

## Introduction

Fetal growth and development are usually monitored using ultrasound. Fetal MRI is complementary to ultrasound in detecting morphologic and functional abnormalities in the fetus (1). Three decades have passed since the first use of MRI in human pregnancy (2). In contradistinction to ultrasound, MRI visualisation of the fetus is not significantly limited by maternal obesity, fetal position, or oligohydramnios, and visualisation of the brain is not restricted by the ossified skull. Through its superior soft tissue contrast resolution, MRI can distinguish individual fetal structures such as the lung, liver, kidney, and bowel. Moreover, MRI provides multiplanar imaging as well as a large field of view, facilitating the examination of fetuses with large or complex anomalies and the visualisation of the lesion within the context of the entire fetal body (3).

Currently, 1.5 Tesla (T) and 3.0 Tesla (T) are being used in antenatal fetal evaluation. It is considered safe for the human fetus; however, some concerns exist at 3.0T with increasing field strengths, and there may be higher tissue heating (4). Safety studies performed at 3.0 T have shown that the specific absorption rate (SAR) generated during fetal imaging is well below the FDA limit, and images obtained at 3.0T demonstrated superior tissue contrast and conspicuity compared to 1.5T (2,5).

DWI is a random Brownian motion of water molecules within a tissue voxel. The apparent diffusion coefficient (ADC) is a quantitative parameter calculated from the DW images that directly reflects the mobility of water molecules in biological tissues (6). While DWI has previously been performed on the brain for an indirect estimate of fetal brain maturation, ADC measurement is reproducible across different gestational ages, and it is now included as a routine in MR examinations in many centers. It greatly contributes to estimating the maturation of other fetal organs like the lung, kidney, and placenta, but it is difficult to estimate the maturation of organs like the liver and spleen due to the low signal of the tissues. It may be used in the future for detecting and describing ischemic lesions or to predict pregnancy outcomes (7), lung maturities (8), kidney maturities (9), and placental insufficiency (10). It is important to understand the ADC values of normal fetal organs at different gestational ages to interpret ADC values in fetal pathologies and compare the normative ADC values to recognise any possible impact of field strength on ADC values on fetal organs at different field strengths. Using terms like brightness will be subjective terminology.

Few studies have reported ADC values of the fetal brain, lung, kidney, and placenta at 1.5T or 3.0T (7, 8, 11-13).

In this study, we aimed to establish and compare the normal reference ADC values of fetal organs and the placenta using 1.5 T and 3.0 T MRI at various gestational ages.

## Methods

**Study population**: This was a retrospective and prospective observational study conducted in a tertiary care hospital. The study was approved by the institutional ethical committee (IEC) (NI/22/APR/82/58), and informed consent was obtained from prospectively included study patients. A total of 103 fetuses between 20 and 38 weeks of gestation were included in each field strength, with a power of 90% and an alpha error of 95%. ADC values from 1.5 T MRI were obtained retrospectively from the MR imaging studies done between January 2018 and March 2021. The ADC values were obtained prospectively from 3T MR imaging studies done between April 2021 and September 2022.

**Inclusion criteria**: Patients referred for the MRI placenta or fetus by a gynaecologist to evaluate suspected fetal anomalies or placental abnormalities on ultrasound who were later found to be normal on MR imaging and follow-up were included in this study.

**Exclusion criteria**: Pregnancies with 1) anomalies 2) Poor image quality/artifacts 3) Placental morphological abnormalities 4) Women with infections and metabolic disorders were excluded.

The follow-up of these fetuses was obtained from the delivery notes and neonatologist examination notes in the hospital database. A telephone conversation with the parents provided further confirmation of the normalcy of the fetuses.

MR Imaging:

All of the 1.5T MRI scans were done with an (Avanto, Siemens, Erlangen, Germany), 8-element torso array coil. All 3T MRI scans were performed using a phased array 16-channel body coil (Signa architect GE, Milwaukee). The patients were positioned feet first in a supine position, and a body coil was placed over the abdomen. The localizer was centred over the anterior superior iliac spine. Our routine imaging protocol included T2 weighted (T2W) SSFSE in 3T (single shot fast spin echo) and HASTE in 1.5T sequence obtained in three orthogonal planes with respect to the fetal brain or trunk (as per the indication) and T1-weighted (T1W) FSPGR in 3T (fast spoiled gradient echo) and FLASH in 1.5T sequence obtained in an axial plane. Diffusion-weighted imaging was performed using single-shot spin-echo-planar imaging (EPI) in the axial plane of the fetal head and trunk with the following parameters: (Table 1).

**Table 1:** MR imaging parameters for the routine sequence and DWI sequence at 1.5T and 3T

MRI	SEQUENCES	TR (ms)	TE (ms)	Slice thickness (mm)	Bandwidth (KHz)	Matrix	FOV (cm)	NEX
1.5 T	AXL T2W HASTE	900	90	4	476	256X194	24-28	1.0
	AXL T1W-FLASH	100	2-5	4	425	256x205	24-28	1.0
	DWI	2000-2500	102	4	965	192X126	24-28	1.0
	b=0, b=800s/mm^2^							
3.0 T	AXL T2W SSFSE	1600-1900	120	4	476	384X300	30	1.0
	AXL T1W-FSPGR	100-150	2-5	4	425	384X300	30-35	1.0
	DWI	2000-2500	88	4	874	256 X 90	30	1.0
	b=0, b=700s/mm							

**Figure 1:**
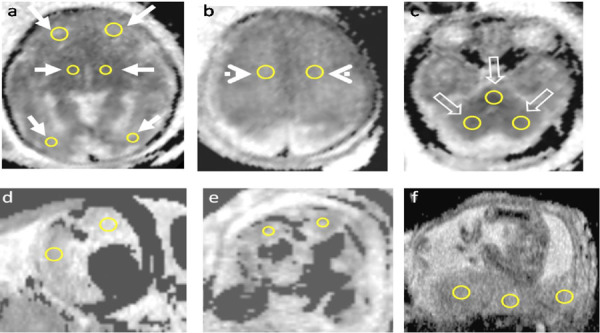
ADC measurements in fetal organs and placenta: Nine ROIs were placed manually in- a) Frontal white matter, Thalamus, Occipital white matter (arrows) b) Centrum semiovale (arrowhead)c) Pons, Cerebellum (open arrows) d) Lungs e) Kidneys f) Placenta

**Post-processing and data analysis**: In 3T MRI, the post-processing of the data was performed on a workstation using Ready View software provided by the manufacturer for DWI with automated generation of ADC maps. In 1.5T MRI, the post-processing of the data was performed on an extended Brilliance workstation. The ADC values were obtained by two radiologists with more than 10 years of experience in fetal imaging. Measurements were standardised according to neuroanatomical landmarks. ROI size ranged from 20 to 40 mm^2^ according to the gestational age and size of the fetal organs. Adjacent structures, such as CSF spaces, were avoided. Regions of interest (ROIs) were manually placed according to the following criteria and the structure of the fetal brain and fetal organs (Figure 1):

**Frontal white matter (FWM)**: measured in the axial plane, at the level of both the frontal horn and the atria.

**Occipital white matter (OWM)**: measured in the axial plane posterior to the atria, at the same slice of the FWM.

**Centrum semiovale (CSO)**: middle of the white matter of the CSO, on the first axial slice, just below the central sulcus and above the ventricle.

**Thalamus**: middle of each thalamus on the axial slice at the level of the basal ganglia.

**Cerebellum**: middle of each cerebellar hemisphere, on the axial slice, at the level of the cerebellar peduncles.

**Pons**: middle of the pons, at mid-fourth ventricle level on the axial plane.

**Lung**: The ROI was placed in the fetal lung parenchyma away from the bronchi in the axial plane.

**Kidney**: The ROI was placed in an axial plane on the fetal cortex, away from the adjacent soft tissue.

**Placenta**: On the axial plane, ROIs were positioned on the lateral portions and on the central portion to measure the average ADC values.

**Statistical analysis**: Statistical analysis was done using the statistical package for the social sciences (SPSS) software. A one-way ANOVA was used to calculate the mean and standard deviation of ADC values. An independent sample t-test was used to compare the ADC values between 1.5T and 3T MRI. Significance was defined as a P-value of less than 0.05.

## Results

**Figure 2:**
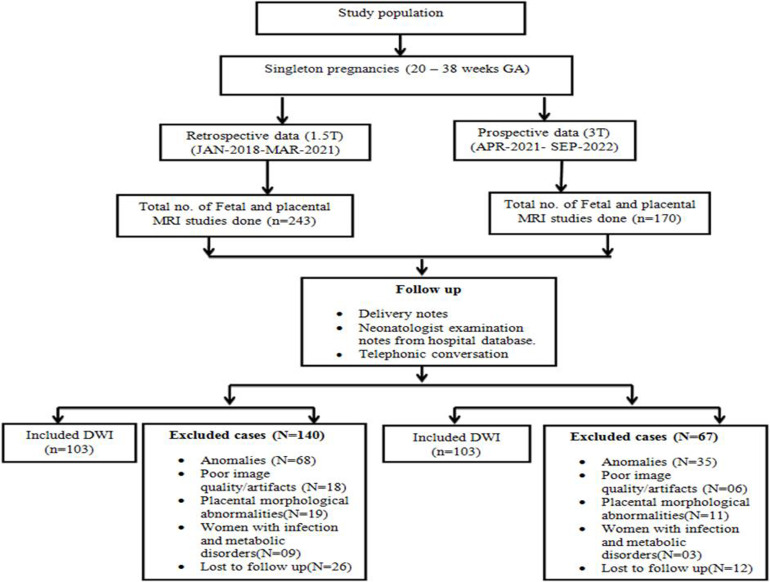
Flow chart for determining study population included in the study

In each field strength, a total of 103 fetuses were separately included from different populations based on gestational age. Retrospectively, a total of 243 fetuses were identified who had imaging at 1.5T, and 140 fetuses were excluded who had anomalies. Prospectively, a total of 170 fetuses were identified who had imaging on 3.0 T; 70 fetuses were excluded who had anomalies (Figure 2). The means and SD of the ADC values of the fetal organs were separated into four gestational age classes, as displayed along with the p-value in Table 2.

**Table 2:** Comparison of mean and standard deviation ADC values of fetal organs and placenta at3T and 1.5TMRI with P-value

Parameters	Weeks	n	(Mean ±SD) (1.5T)	N	(Mean ±SD) (3.0T)	p-value
Frontal white	20-24	32	1.58±0.04	51	1.59±0.05	
matter (FWM)	25-29	24	1.64±0.03	19	1.66±0.04	
	30-34	32	1.66±0.56	22	1.69±0.04	
	35-38	15	1.71±0.06	11	1.76±0.09	
	**20-38**	**103**	**1.64±0.06**	**103**	**1.64±0.08**	0.64
Occipital white	20-24	32	1.58±0.06	51	1.60±0.06	
matter (OWM)	25-29	24	1.67±0.06	19	1.67±0.02	
	30-34	32	1.65±0.05	22	1.66±0.03	
	35-38	15	1.66±0.11	11	1.65±0.03	
	**20-38**	**103**	**1.64±0.07**	**103**	**1.64±0.07**	0.60
Centrum semi	20-24	32	1.61±0.06	51	1.59±0.02	
ovale	25-29	24	1.66±0.06	19	1.63±0.01	
	30-34	32	1.65±0.05	22	1.64±0.02	
	35-38	15	1.66±0.05	11	1.67±0.02	
	**20-38**	**103**	**1.65±0.05**	**103**	**1.65±0.05**	< 0.001
Pons	20-24	32	1.23±0.04	51	1.28±0.08	
	25-29	24	1.22±0.07	19	1.21±0.06	
	30-34	32	1.20±0.05	22	1.19±0.08	
	35-38	15	1.18±0.05	11	1.18±0.07	
	**20-38**	**103**	**1.21±0.05**	**103**	**1.21±0.05**	< 0.001
Thalamus	20-24	32	1.31±0.05	51	1.24±0.06	
	25-29	24	1.28±0.05	19	1.23±0.05	
	30-34	32	1.25±0.03	22	1.15±0.06	
	35-38	15	1.23±0.02	11	1.16±0.06	
	**20-38**	**103**	**1.27±0.05**	**103**	**1.21±0.07**	< 0.001
Cerebellum	20-24	32	1.42±0.06	51	1.39±0.12	
	25-29	24	1.42±0.05	19	1.35±0.08	
	30-34	32	1.38±0.05	22	1.31±0.07	
	35-38	15	1.37±0.05	11	1.32±0.08	
	**20-38**	**103**	**1.40±0.06**	**103**	**1.36±0.10**	< 0.001
Lungs	20-24	32	1.89±0.10	51	1.82±0.08	
	25-29	24	1.96±0.15	19	1.94±0.10	
	30-34	32	2.08±0.10	22	2.05±0.15	
	35-38	15	2.13±0.05	11	2.07±0.15	
	**20-38**	**103**	**2.00±0.14**	**103**	**1.92±0.15**	0.101
Kidney	20-24	32	1.36±0.06	51	1.33±0.10	
	25-29	24	1.34±0.05	19	1.37±0.08	
	30-34	32	1.28±0.06	22	1.31±0.09	
	35-38	15	1.27±0.06	11	1.33±0.08	
	**20-38**	**103**	**1.32±0.07**	**103**	**1.34±0.09**	< 0.001
Placenta	20-24	32	1.91±0.08	51	1.84±0.05	
	25-29	24	2.02±0.07	19	1.96±0.04	
	30-34	32	2.05±0.04	22	2.05±0.04	
	35-38	15	2.11±0.07	11	2.15±0.05	
	**20-38**	**103**	**2.01±0.10**	**103**	**1.94±0.11**	< 0.001

The mean ADC values of the cerebral white matter areas were significantly higher than the deep grey areas. Statistically significant differences were observed between the two field strengths in all parameters except for FWM, OWM, and kidneys. The intra-observer variability was 1%, and the inter-observer variability was 2%.

The ADC values of fetal organs and placenta acquired in the present study at 3.0T were compared with the ADC values suggested by other studies (Moradi B et al. (11), Afacan O et al. (8) at 3T from 28 to 38 weeks, which showed a statistically significant difference and slightly lower values (Table 3).

**Table 3:** Comparison of mean and standard deviation values of fetal brain and organs of present study (3T MRI) with mean and standard deviation reference values of **Behnazmoradi *et.al.* (11) and OnurAfacan *et al.* (8)** study (3T MRI)

Parameters	Present study 3T (mean ±SD) (28-38weeks) n=103, b=700	Behnazmoradi et al. (11) 3T (mean ±SD) (28-38weeks) n=18, b=700	P-value
Frontal white matter (FWM)	1.64±0.082	1.74± 0.13	
Occipital white matter (OWM)	1.64±0.066	1.75± 0.17	
Centrum semiovale (CSO)	1.62±0.038	1.69 ± 0.12	
Pons	1.23±0.092	1.10 ± 0.10	
			<0.001
Thalamus	1.21±0.070	1.27 ± 0.15	
Cerebellum	1.36±0.106	1.28± 0.18	
Lungs (OnurAfacan et al. (8))	1.92±0.151	2.46±0.23	
Kidneys	1.34±0.09	-	
Placenta	1.94±0.118	-	

ADC values of fetal organs and placenta acquired in the present study at 1.5T and ADC values suggested by other studies Boyer AC et al. (12), Arthurs OJ et al. (7), and Razek AA et al. (13)) at 1.5T, which showed a statistically significant difference and slightly higher values (Table 4).

**Table 4:** Comparison of mean and standard deviation values of fetal brain and fetal organs of present study (1.5T MRI) with mean and standard deviation reference values of A.C. Boyer et al. (12) ,0. J. Arthurs et al. (7) and Razek AA et al. (13) studies at 1.5T

Parameters	Present study 1.5T(mean ±SD) n=103, b=800	A.C. Boyer et al. (12) 1.5T (mean ±SD) n=50, b=1000	Arthurs et al. (7) (mean ±SD) n=24, b=700	Razek AA et al. (13) (mean ±SD) n=10, b=1000	P-Value
**FWM**	1.64±0.067	1.37 ± 0.2	2.17±0.22	-	<0.001
**OWM**	1.64±0.079	1.34 ± 0.25	2.01±0.19	-	<0.001
**CSO**	1.65±0.052	1.36 ± 0.29	1.97±0.23	-	<0.001
**Pons**	1.21±0.053	1.05 ± 0.17	0.94±0.12	-	<0.001
**Thalamus**	1.27±0.057	1.06 ± 0.19	1.13±0.10	-	<0.001
**Cerebellum**	1.40±0.062	1.26 ± 0.20	1.65±0.13	-	<0.001
**Lung**	2.00±0.144	-	-	1.92 ± 0.3	<0.001
**Kidney**	1.32±0.072	-	-	1.47 ± 0.1	<0.001
**Placenta**	2.01±0.101	-	-	1.59 ± 0.1	<0.001

**ADC values versus Gestational Age**: White-matter regions showed a positive and significant correlation with increasing gestational age in both field strengths. A statistically weak negative correlation was observed between increasing gestational age and ADC measurements obtained in the thalamus (r = -0.53 at 1.5T and r = -0.43 at 3T), cerebellum (r = -0.356 at 1.5T and r =-0.27 at 3T), pons (r = -0.316 at 1.5T and r =-0.51 at 3T), and kidney (r = -0.52 at 1.5T and r =-0.57 at 3T). Lung (r = 0.68 at 1.5T and r = 0.69 at 3T) and placenta (r = 0.69 at 1.5T and r = 0.93 at 3T) also showed a positive and significant correlation with increasing gestational age in both field strengths.

## Discussion

DWI is a random Brownian motion of water molecules within a tissue voxel. The apparent diffusion coefficient (ADC) is a quantitative parameter calculated from the DWI that directly reflects the mobility of water molecules in biological tissues (6). This study is to establish and compare the normal reference ADC values of fetal organs and the placenta using 1.5 T and 3.0 T MRI at various gestational ages. A total of 103 fetuses between 20 and 38 weeks of gestation were included in both field strengths.

Similar to other studies in normal fetuses (6,14-16), in the present study at both 1.5T and 3.0T, we also found that with increasing gestational age, ADC linearly decreased in the Thalamus, Cerebellum, and Pons, reflecting the morphological changes of brain maturation that involve increasing cellularity, neuronal maturation, and myelination (14).

Hoffmann C. et al. (14) stated in their study that ADC was found to be significantly higher in the white matter region than in the grey matter regions (thalamus, pons, and cerebellum), suggesting an immature status of fibre myelination during fetal development. The present study also proved the same results in both 1.5T and 3T: the ADC values were significantly higher in the white matter region than in deep grey matter regions.

The present study provides average ADC values of the lung from 20 to 38 weeks at 1.5T and 3.0T (2.00±0.144× 10^−3^ mm^2^ /s and 1.92±0.15× 10^−3^ mm^2^ /s, respectively). The results show that ADC values correlate with increasing gestational age when b-values were acquired (b = 0 and b = 700,800) in 103 subjects on a 1.5T and 3T MRI. The ADC values obtained by the present study were slightly lower than the reported ADC lung values. Manganaro et al. (17) showed a high correlation between gestational age and ADC when 3 b-values (b = 0, 250, and 750) were acquired in 50 subjects, and the mean ADC was (2.352±0.6210^−3^ mm^2^/s). Afacan O et al. (8) conducted a study to evaluate the feasibility of using diffusion-weighted magnetic resonance imaging (DW-MRI) to assess the fetal lung apparent diffusion coefficient (ADC) at 3 Tesla in 71 subjects, and the results suggested that the mean ADC value ranged from (1.89>10^−3^ mm^2^/s to 2.46×10^−3^ mm^2^/s) and increased with GA.

Witzani et al. (9) found no significant correlation between gestational age and ADC of a kidney when ADC was measured using a two-b-value diffusion acquisition (b = 0 and b = 1000) from 16 to 38 weeks ((0.74–1.65 10^−3^mm^2^/s) in 218 subjects on a 1.5T scanner. Compared to this study, the present study provides average ADC values from 20 to 38 weeks (1.32±0.072 and 1.34±0.06×10^−3^mm^2^/s). There was a negative correlation between gestational age and ADC of the kidney, which is not statistically significant, when b-values were acquired (b = 0 and b = 700,800) in 103 subjects on a 1.5T and 3T MRI. The ADC values obtained by the present study are slightly lower than the reported ADC kidney values.

Manganaro et al. (10) showed a correlation between gestational age and ADC of the placenta when low b-values were acquired (b = 0, 200, 700) in 145 subjects; the mean ADC was 1 to 2.4 mm^2^/s, whereas ADC values calculated from high b-values (b = 50, 200, 700) showed an inverse correlation with GA in 50 subjects; the mean ADC was 1.5–1.7 mm^2^/s. Compared to this study, the present study provides average ADC values of the placenta from 20 to 38 weeks at 1.5T and 3.0T (2.01±0.101× 10^−3^ mm^2^/s and 1.94 ± 0.118× 10^−3^ mm^2^/s, respectively). The results show that ADC values correlate with increasing gestational age when b-values were acquired (b = 0 and b = 700,800) in 103 subjects on a 1.5T and 3T MRI. The ADC values obtained by the present study were slightly lower than the reported placental ADC values.

In this study, the average ADC values of fetal organs and the placenta were measured in two different strengths of magnetic fields. The results of this study showed that the ADC values of 1.5T and 3T MRI are significantly different. This might seem strange at first because, in theory, most scanners automatically create the ADC map voxel by voxel, and the value of each voxel shows how the logarithm of the signal intensity relates to the b value. The calculated ADC value is independent of magnetic field strength (2,18). A possible explanation for the significant difference in ADC of different field strengths is the difference between the technique and parameters applied on the 3T and 1.5T magnets, two different b-values, different vendors, and different study populations, which results in statistical significance even with small differences in measurements. Lavdas I et al. (19) also found that measured ADC is significantly different between the two fields and between the same field strength across the two vendors.

The measurements of the normal apparent diffusion coefficient (ADC) at 1.5T and 3T MRI in different fetal brain regions and organs can help to better understand the aetiology and evolution of certain pathologies like demyelination, cerebral infection, metabolic disorders, lung and renal maturities, placental insufficiency, and FGR (fetal growth retardation) (20-22).

The relatively small sample size is the primary limitation of this study. Second, though we used respiratory gating, there can still be movement due to maternal and fetal causes that are not compensated. Third, we didn't include the same fetuses for the 1.5T and 3T examinations because this would have exposed the fetus to unnecessary MRI examinations. The retrospective nature of the study is also a limitation. Another limitation is the slight difference between the technique and parameters applied to the 3T and 1.5T magnets.

We recommend further studies be conducted with a larger sample size with the same population and technique in both field strengths.

This study gives the reference values for both 1.5T and 3T MRI of vital organs. We found a negative association between ADC and gestational age in fetal brain regions (thalamus, pons, and cerebellum) and the fetal kidney. Statistically significant differences were observed between the two field strengths in all parameters except for FWM, OWM, and kidneys. The current study shows that diffusion-weighted MRI can offer a promising technique to evaluate the structural development of fetal organs and can potentially act as a biomarker for predicting the functionality of the fetal organs in abnormalities. In the future, it could also be useful to study changes related to demyelination, cerebral infection, metabolic disorders, placental insufficiency, and FGR (fetal growth retardation), where MRI can be normal at the initial stages.
